# Metal/Si Nanowire Arrays for Hot Electron Catalysis

**DOI:** 10.1002/smll.74983

**Published:** 2026-08-02

**Authors:** Theresa Bartschmid, Furkan Atalay, Maurizio Musso, Amin Farhadi, Johannes Menath, Maret Ickler, Nicolas Vogel, Gilles R. Bourret

**Affiliations:** ^1^ Department of Chemistry and Physics of Materials University of Salzburg Salzburg Austria; ^2^ Institute of Particle Technology Friedrich‐Alexander University Erlangen‐Nürnberg Erlangen Germany

**Keywords:** Au nanoparticles, hot electron, metal‐assisted chemical etching, plasmon‐driven catalysis, SERS, silicon nanowires

## Abstract

The application of high–refractive index substrates to boost near‐field enhancements in plasmonic nanostructures for photocatalysis remains limited. Here, we combine hexagonally‐arranged vertically‐aligned silicon nanowire (VA‐SiNW) arrays with Au nanoparticles and Au/Ag nanoshells to study the plasmon‐driven dimerization of p‐nitrothiophenol (p‐NTP) into 4,4′‐dimercaptoazobenzene (DMAB). In situ Raman spectroscopy reveals that VA‐SiNWs can enhance local electric fields at the metal nanostructures, which we use for SERS and hot electron catalysis. Despite producing the strongest near‐field enhancement and the highest SERS response, AuNPs/SiNW arrays do not deliver the highest reaction rates, indicating that near‐field intensity alone does not govern catalytic performance. This discrepancy is attributed to steric hindrance within highly confined hotspots at the Au/Si interface. Compared with the Au‐based substrates, Ag/SiNW arrays show higher photocatalytic activity, with 532 nm excitation dramatically accelerating p‐NTP photoreduction. Ag/SiNW arrays also photocatalyze 4‐mercaptobenzoic acid decarboxylation, demonstrating their ability to drive oxidation reactions via the generation of reactive oxygen radicals. Overall, our results indicate that VA‐SiNW arrays can serve as high‐surface‐area photonic supports for plasmon‐driven chemistry and highlight the critical interplay between field enhancement, hotspot accessibility, and metal choice in optimizing hot electron‐induced photocatalytic performance.

## Graphical abstract

Silicon nanowire arrays enhance plasmon‐driven catalysis by strengthening light–matter interactions on Au and Ag nanostructured coatings. Hotspot accessibility, rather than near‐field intensity alone, governs photocatalytic performance, while Ag nanoshells exhibit superior activity. The work establishes high‐surface‐area Si nanowire arrays as versatile platforms for hot‐electron and reactive oxygen species photocatalysis.

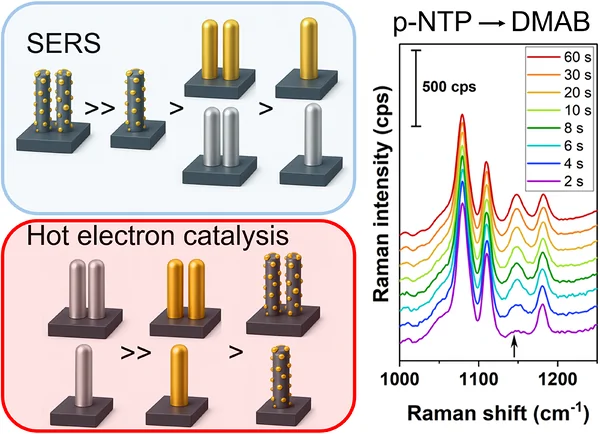

## 1 Introduction

The growing demand for cost‐effective and sustainable energy highlights the increasing need to replace fossil fuels and find alternative paths to producing materials. Plasmonic photocatalysis addresses this challenge by harnessing solar energy to drive chemical reactions [1, 2]. Under light irradiation, metal nanoparticles (NPs) can sustain localized surface plasmon resonance (LSPR), causing collective oscillations of the free electrons at the metal NP surface [3, 4]. LSPR excitation can result in very large enhancements of the electric near‐field (E‐field) and light absorption, both of which have been used to drive photochemical processes on metal catalysts [3, 4, 5, 6]. Excited LSPRs can generate energetic electron‐hole pairs that relax within a few hundred femtoseconds via electron‐electron scattering to yield a Fermi‐Dirac distribution of “hot” charge carrier energies with an elevated temperature [2, 7, 8, 9]. During both of these stages, hot electrons and holes can be transferred to molecules adsorbed at the metal surface, and drive chemical reactions [7, 9, 10, 11, 12]. In the presence of oxygen or water, hot charge carriers can generate reactive oxygen species (ROSs), such as oxygen superoxide O_2_˙^−^ and hydroxyl radicals HO˙, which can often mediate oxidation reactions [13, 14, 15, 16]. After a few picoseconds, the hot charge carriers are scattered by phonons, which leads to photothermal heating of the metal NP [2, 7, 8]. To date, hot electron transfer, the generation of ROSs, and photothermal heating have been considered to explain enhanced reaction rates and/or selectivity observed during plasmonic excitation of nanostructured metal catalysts [7, 9, 13, 14, 15, 16, 17, 18, 19]. All three processes require high E‐field enhancements, either within the metal or at the metal surface, typically achieved by engineering electromagnetic hotspots around, or in‐between plasmonic nanostructures [3, 20, 21].

To investigate the physics involved in plasmon‐assisted catalysis, model reactions that can be sensitively tracked under plasmonic excitation are frequently employed. Among them, the dimerization of p‐nitrothiophenol (p‐NTP) to form 4,4′‐dimercaptoazobenzene (DMAB) is a widely studied reaction in plasmonic photocatalysis, because it is relatively well‐understood and can be monitored in situ within a Raman microscope using the surface‐enhanced Raman scattering (SERS) effect [7, 9, 17]. p‐NTP can be reduced to DMAB via the following reduction reaction (Figure 1), which involves 8 electrons [9, 22, 23, 24, 25, 26, 27].

**FIGURE 1:**
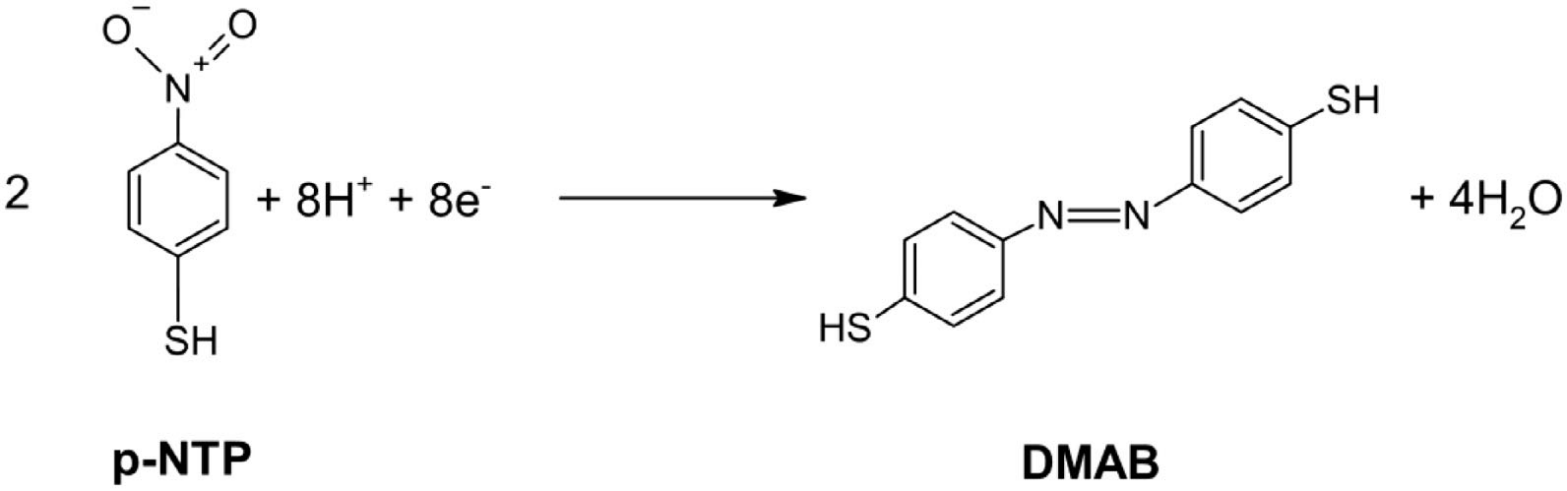
Reaction of p‐NTP to form DMAB. Reduction reaction of p‐nitrothiophenol (p‐NTP) to 4,4′‐dimercaptoazobenzene (DMAB) based on literature [22, 24, 26].

Light can replace an electrochemical potential to cathodically charge a plasmonic nanostructure with hot electrons, which can then reduce adsorbed p‐NTP molecules into DMAB. This hot electron reduction requires: (i) an adequate mobility of p‐NTP molecules (diffusion and rotational movements) on the bare metal surface to achieve a head‐to‐head configuration, (ii) a charge transfer from the metal to the p‐NTP molecules, (iii) a high surface coverage of the p‐NTP at the metal surface, and (iv) a metal with suitable plasmonic properties, i.e. large E‐field enhancement at the wavelength used, and activity toward the targeted reaction [12, 22, 27, 28, 29, 30].

This reaction has been reported to occur predominantly in electromagnetic hotspots, which are typically generated by introducing nanoscale gaps between metal nanoparticles—a strategy that is synthetically difficult to control [9, 31, 32, 33, 34]. Alternatively, anisotropic nanostructures with sharp features can produce effective hotspots to drive this catalytic reaction, but their fabrication is even more demanding [35, 36]. Finally, coupling metal nanoparticles with high–refractive index dielectric nanostructures provides another route to generate intense electromagnetic hotspots [31, 37, 38, 39]. In that regard, hexagonally arranged, vertically‐aligned silicon nanowire arrays (VA‐SiNW arrays) have been particularly effective at enhancing the near E‐field when coated with AuNP monolayers [31, 37, 38, 40]. VA‐SiNWs can resonantly interact with light via the excitation of diameter‐dependent waveguiding modes and length‐dependent Fabry‐Pérot resonances to increase the E‐field at the silicon surface [31, 37, 41, 42, 43, 44, 45]. When coated with metal NPs, these arrays can generate large E‐field enhancements within and at the metal NP surface [31, 37, 40, 46]. Such VA‐SiNWs can be conveniently fabricated using a combination of colloidal lithography and metal‐assisted chemical etching (MACE), avoiding the need for nanofabrication tools and cleanroom infrastructure [31, 37, 41, 43, 47, 48]. The decoration with AuNPs can subsequently be realized using wet‐chemical methods [31, 37]. The approach is appealing because it does not require the use of complex nanofabrication to form well‐defined hotspots. Additionally, the methodology is compatible with the preparation of macroscopic, centimeter‐scale substrates and is potentially compatible with E‐field enhancement on all types of metal NPs, as long as the metal NPs can be deposited on the silicon surface. Finally, by adjusting the conditions used to perform the colloidal lithography, it is possible to synthesize either isolated SiNW arrays, i.e., monomers, or SiNW dimer arrays, where most SiNWs are present as SiNW dimers with sub‐50 nm gaps [37, 49]. Our group recently demonstrated that VA‐SiNW dimer arrays can enhance the E‐field intensity in the SiNW dimer gap region even further, which was used to maximize the SERS signal [37]. However, the use of such metal/VA‐SiNW hybrid arrays for plasmonic photocatalysis has not yet been demonstrated.

Herein, we report the systematic investigation of hexagonally arranged VA‐SiNW arrays with gold and silver nanostructured coatings for the plasmonic conversion of p‐NTP to DMAB. Arrays composed of VA‐SiNW monomers and dimers were coated with either colloidal AuNP monolayers or with uniform Au or Ag layers to form a defined nanoshell covering the entire nanowire surface. The activity of the prepared metal/Si nanostructured arrays for the photodimerization of p‐NTP to form DMAB was investigated using in situ Raman spectroscopy with a confocal microscope. The formation of new Raman bands can be observed during DMAB formation, associated with the newly formed N═N double bond [7, 9, 50, 51].

## 2 Results and Discussion

### 2.1 Synthesis of Vertically‐Aligned Au‐ and Ag‐SiNW Arrays

Figure 2 gives an overview of the fabrication of the different nanostructured substrates used in this study. First, the VA‐SiNW scaffolds are synthesized by MACE. In brief, a colloidal monolayer deposited onto a Si wafer is used as a mask to create a gold nanohole array (Figure 2a; Au is yellow in Figure 2) [52].

**FIGURE 2:**
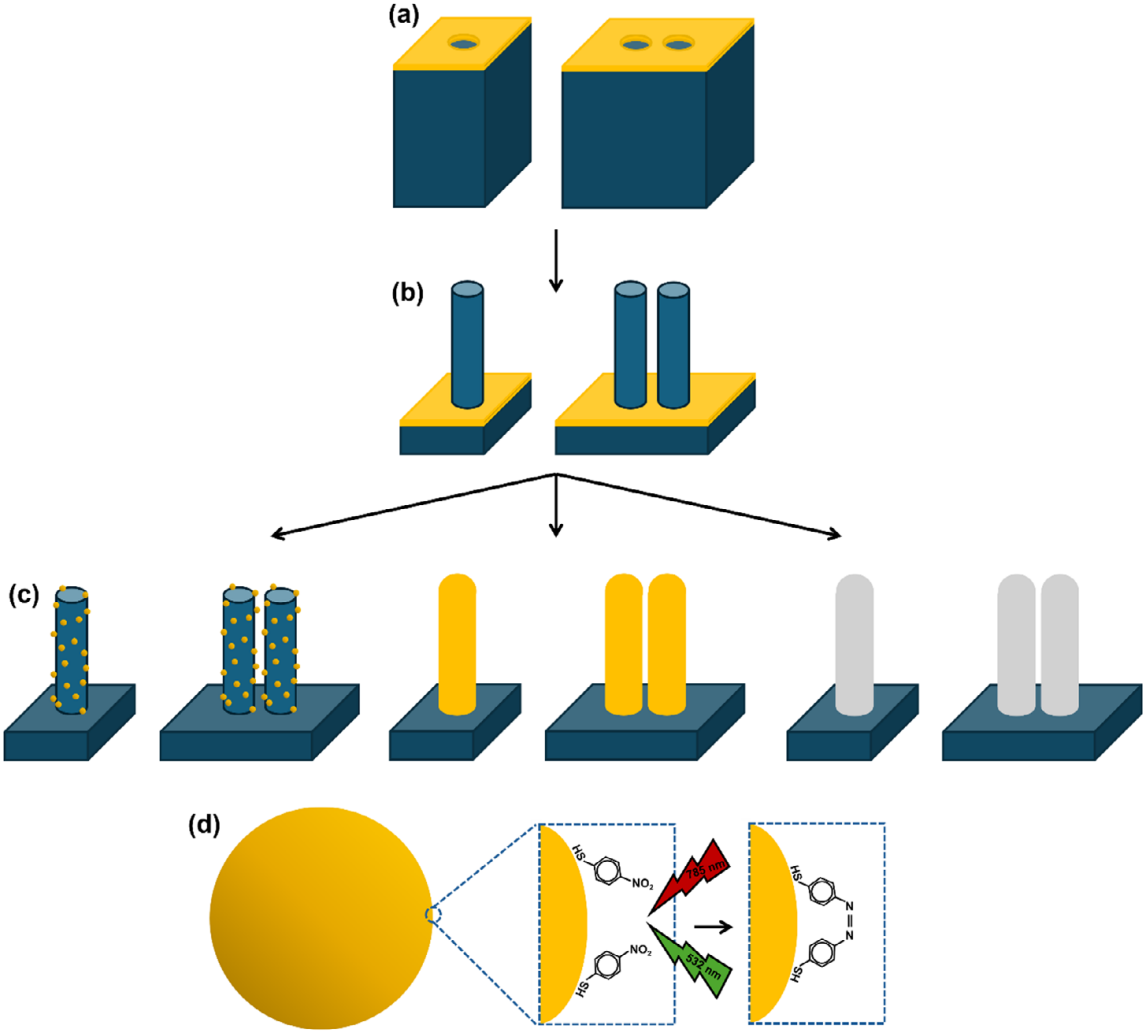
Fabrication of the different metal‐SiNW arrays. (a) A Au nanomesh produced via colloidal lithography acts as a catalytic mask. (b) During MACE, the Au nanomesh film sinks into the substrate, producing a vertically aligned SiNW array. (c) After dissolution of the catalytic Au film, the SiNWs are decorated with Au and Ag nanostructures and covered with p‐nitrothiophenol (p‐NTP). (d) The photodimerization reaction is induced and studied via Raman spectroscopy.

This is achieved by sputtering a thin adhesion layer composed of aluminum‐doped zinc oxide and a Au thin film with approximately 30–40 nm thickness on top of the colloidal monolayer. The removal of the colloidal template spheres using adhesive tape results in a continuous metal nanomesh at the silicon surface, with dimensions defined by the templating colloidal particles (Figure 2a) [53]. Next, MACE is performed in an aqueous solution of hydrogen peroxide and hydrofluoric acid [48]. During MACE, the silicon is directionally etched at the Au/Si interface, producing the VA‐SiNW arrays (Figure 2b) [48]. The SiNW length is controlled by the MACE duration, set to 2 min, which leads to SiNWs with average lengths of (867 ± 25) nm.

After dissolution of the Au nanomesh film, the different Au and Ag nanostructured coatings were synthesized via (i) self‐assembly of pre‐synthesized citrate‐stabilized AuNPs on VA‐SiNW arrays functionalized with amino groups creating nanoparticle‐decorated nanowires, referred to as AuNPs/SiNW arrays [31, 37], and by conformally coating the VA‐SiNWs via DC sputtering with either (ii) a thin layer of Au (Au nanoshell/SiNWs) or (iii) a thin layer of Ag (Ag nanoshell/SiNW arrays) to produce arrays of metal nanoshells coated on top of the nanowires [31, 37, 41, 42, 43]. Using this synthetic platform, we create a library of different plasmonic substrates to systematically explore structure‐property relationships governing photocatalysis. Specifically, we examine the effects of coating morphology (nanoparticle vs. continuous coating), plasmonic metal (Au vs. Ag), SiNW array configuration (monomer vs. dimer arrays), and wavelength (785 vs. 532 nm).

Scanning electron microscopy (SEM) images of the fabricated structures are given in Figure 3. The colloidal AuNPs used to decorate the VA‐SiNWs had an average size of (19 ± 2) nm. The thickness of the Au nanoshell prepared via sputtering was (17 ± 2) nm, as determined via scanning transmission electron microscopy (STEM) dark‐field analysis and via STEM‐energy dispersive X‐ray spectroscopy (EDX) analysis, whereas the thickness of the Ag nanoshell was slightly larger, at (24 ± 2) nm. STEM‐EDX maps are shown in Figure S1. Individual AuNPs with a low degree of aggregation can be clearly seen on the AuNPs/SiNW arrays (Figure 3a,d). As previously demonstrated, the AuNPs also enter the gap region of the SiNW dimer arrays (Figure 3d) [37]. The metal nanoshells are instead conformal with a structured but relatively smooth surface and rounded edges at the top (Figure 3b,c,e,f). Additionally, some metal/SiNW dimers feature fully closed gaps due to the gap‐size distribution generated during colloidal lithography [37, 49].

**FIGURE 3:**
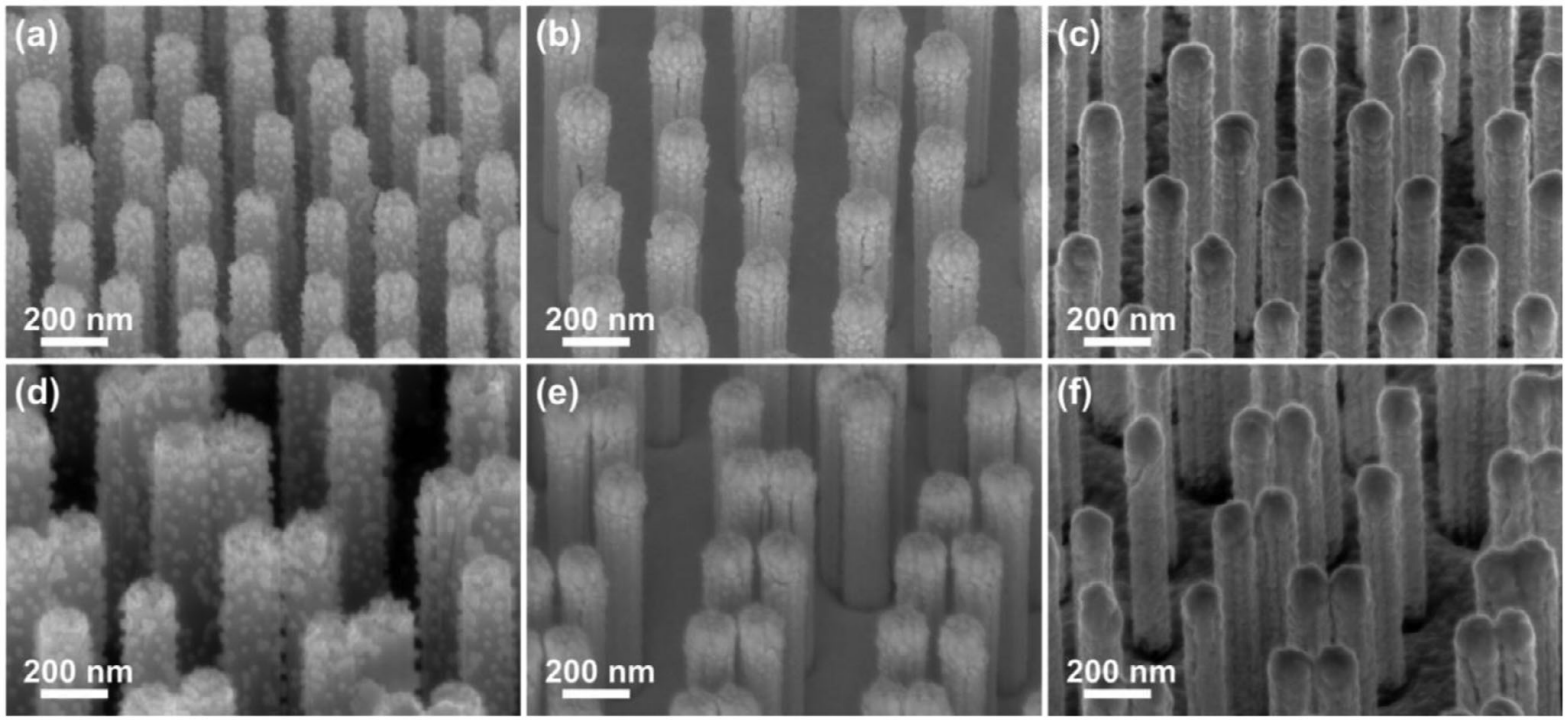
Metal‐SiNW arrays. SEM images of the fabricated Au and Ag morphologies on vertically aligned SiNWs. (a,d) Self‐assembled AuNPs on functionalized SiNW monomer (a) and dimer (d) arrays. (b,e) Au nanoshells with an average thickness of (17 ± 2) nm around SiNWs after 1000 s of sputtering on SiNW monomer (b) and dimer (e) arrays. (c,f) Ag nanoshells with an average thickness of (24 ± 2) nm around SiNWs after 500 s of sputtering on SiNW monomer (c) and dimer (f) arrays.

### 2.2 Raman Measurements and SERS Effect

Raman measurements were performed at a laser excitation wavelength of 785 nm for both the Au‐ and Ag‐coated VA‐SiNW arrays using a laser power of 1 mW (2 s exposure time and 3 accumulations), which is low enough to avoid DMAB formation (see Figure S2). In addition, the Ag nanoshell/VA‐SiNW substrates were irradiated with a 532 nm laser at the lowest power available, i.e., 0.1 mW (1 s exposure time and 2 accumulations). Full Raman spectra before any light‐induced reaction of p‐NTP are shown for all AuNPs, Au nanoshell and Ag nanoshell/VA‐SiNW dimer arrays in Figure 4a together with a SERS spectrum recorded on a flat Si substrate decorated with AuNPs. The respective spectra for the monomer arrays can be found in Figure S3. Figure 4b gives an overview of the Raman band intensity at ca. 1340 cm^−1^ (corresponding to the NO_2_ symmetric stretching of p‐NTP) [7, 9, 10, 11, 12] for the different metal/SiNW monomer and dimer arrays. AuNPs assembled on flat Si are included as a reference system to identify the role of the nanostructured silicon substrate in enhancing Raman signals.

**FIGURE 4:**
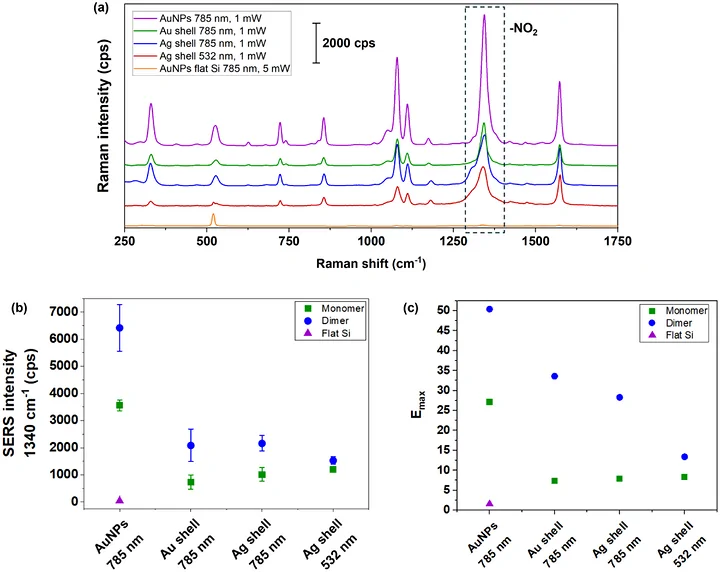
SERS results. (a) Offset SERS spectra of p‐NTP recorded on vertically aligned SiNW dimer array substrates with different metal nanostructures as indicated in the Figure legend. Additionally, the SERS spectrum of the reference sample (AuNPs on flat Si) is shown, recorded at 5 mW with all other measurement parameters kept constant. (b) SERS intensity for the Raman band at 1340 cm^−1^ corresponding to the NO_2_ symmetric stretching vibration of p‐NTP before the dimerization reaction at 785 nm laser excitation, unless stated otherwise. (c) Simulated maximum enhancement of the E‐field strength E_max_ on the different substrates. Au and Ag shells were simulated with a thickness of 17 nm, yielding a gap size of 6 nm between the dimers [7, 9, 10, 11, 12].

The measured SERS intensities are highest for the self‐assembled AuNPs/SiNW arrays. This is attributed to the large E‐field enhancement present at the Au‐Si interface in the AuNPs/SiNW monomer and dimer substrates, which is maximized for AuNPs/SiNW dimer arrays [37]. Figure 4c shows the maximum E‐field strength enhancements derived from finite‐difference time‐domain (FDTD) simulations, which qualitatively agree with the measured SERS signals. This is also visible on the simulated E‐field enhancement maps (Figure 5), showing a higher field enhancement around the AuNPs located on the SiNW surface (Figures 4c and 5c–g) [54].

**FIGURE 5:**
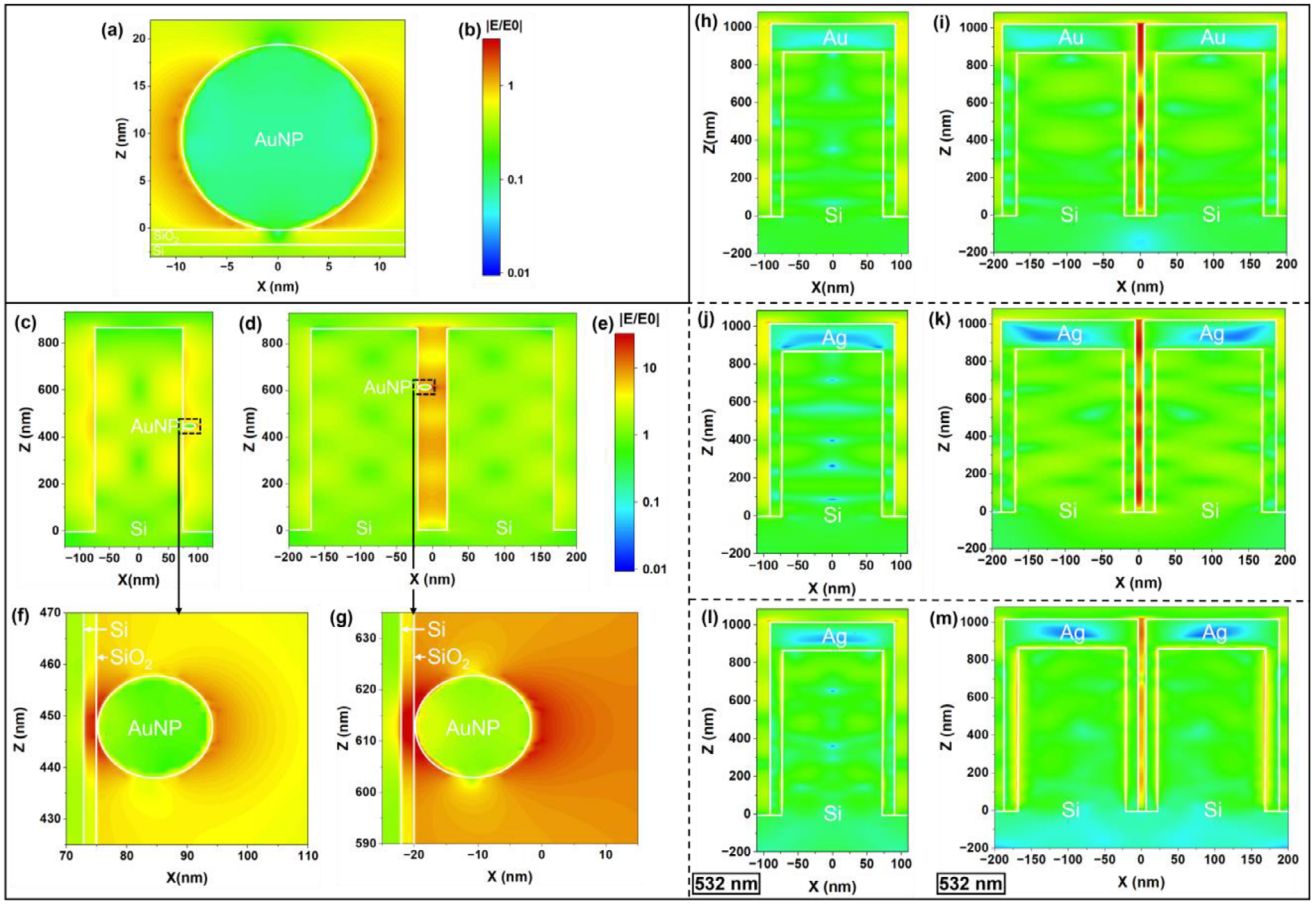
Simulated E‐field enhancement maps. (a,b) An AuNP on a flat Si substrate and the corresponding color scale. (c–g) AuNPs/SiNW substrates at 785 nm excitation. (c,f) AuNPs/SiNW monomer, and (d,g) AuNPs/SiNW dimer arrays. (e) Color scale bar used for the E‐field enhancement maps shown in (c‐m). (h,i) Au nanoshell/SiNW substrates at 785 nm excitation. (h) Au nanoshell monomer and (i) Au nanoshell dimer. (j–m) Ag nanoshell/SiNW substrates at 785 nm excitation (j,k) and 532 nm excitation (l,m). (j,l) Ag nanoshell monomer, and (k, m) Ag nanoshell dimer.

Additionally, the metal/SiNW dimer arrays consistently perform better compared to the metal/SiNW monomer arrays at both 785 nm and 532 nm, which confirms our previous findings on AuNPs/SiNW dimer arrays [37]. At both wavelengths, the maximum E‐field strength enhancement *E*_max_ is higher for the dimer arrays compared to the VA‐SiNW monomer arrays (Figures 4c and 5). Based on the high *E_max_* values simulated for the Au nanoshell and Ag nanoshell/SiNW dimer arrays, slightly higher SERS intensities would have been expected on these substrates (Figures 4c and 5i,k). We attribute this discrepancy to geometrical inhomogeneities in the dimer arrays: These dimer substrates show a considerable distribution of gap sizes due to experimental limitations, causing some of the Au and Ag nanoshells to overlap rather than forming a gap. Additionally, the rounded edges of the metal nanoshells (see Figure 3) are expected to produce slightly lower *E_max_* values, compared to the sharp edges used in the FDTD simulations [55]. As a reference, a AuNP on a flat Si surface produced a maximum field strength enhancement that is ca. one order of magnitude lower compared to the metal/SiNW substrates (Figures 4c and 5a,b), explaining the low Raman signals obtained on the reference substrate.

Finally, the similar SERS intensities measured on the Ag nanoshell/SiNW monomer and dimer arrays at 532 nm indicate a less efficient coupling between adjacent Ag nanoshell/SiNWs in the dimers at 532 nm, compared to 785 nm. This is in qualitative agreement with our FDTD simulations that show a similar E‐field enhancement at 532 nm on both Ag nanoshell/SiNW monomers and dimers (Figures 4c and 5j–m).

### 2.3 Plasmon‐Driven Dimerization of p‐NTP

Having established that SiNW dimer arrays outperform SiNW monomer arrays, and that AuNPs/SiNWs provide the largest near‐field enhancements for SERS, we now evaluate the performance of the same substrates in plasmon‐driven catalysis. In situ Raman spectroscopy was used to evaluate the performance of the different substrates for the photo‐induced dimerization of p‐NTP (Figure 1). To this end, the Raman intensities of the 1340 cm^−1^ band, which corresponds to the symmetric NO_2_ stretching of the parent p‐NTP molecule, and the 1140 cm^−1^ band, which indicates the formation of DMAB, were compared as a function of irradiation time (Figure 6). The exact origin of this band is still under debate. However, recent studies used theoretical DFT calculations to assign the band to a combination vibration of the symmetrical C─N stretching and the C─H scissoring vibrations [10, 56]. These two bands are least influenced by other bands in the Raman spectrum and thus allow for following the p‐NTP dimerization reaction most accurately [7, 9, 10, 11, 12, 57]. For all experiments, the efficiency of the different metal/Si substrates toward the photodimerization of p‐NTP was evaluated by comparing the ratio of band intensities I_1140_/I_1340_, where I_1140_ and I_1340_ are the Raman intensities of the 1140 cm^−1^ (DMAB) and 1340 cm^−1^ (p‐NTP) bands. The band intensities were obtained after peak deconvolution and integration of the area below the respective bands (see Section 4 for details). This intensity ratio acts as an internal reference, normalizing the photodimerization results to the total number of molecules. In the systematic design of the samples tested, the metal surface area varies between substrates, and therefore the total number of probed molecules during the Raman measurements is not constant, which complicates a direct comparative analysis. This intensity ratio enables us to compare the different substrates and reduces the influence of substrate‐to‐substrate differences in SERS enhancement as well as potential baseline shifts within the measurement series on one substrate.

**FIGURE 6:**
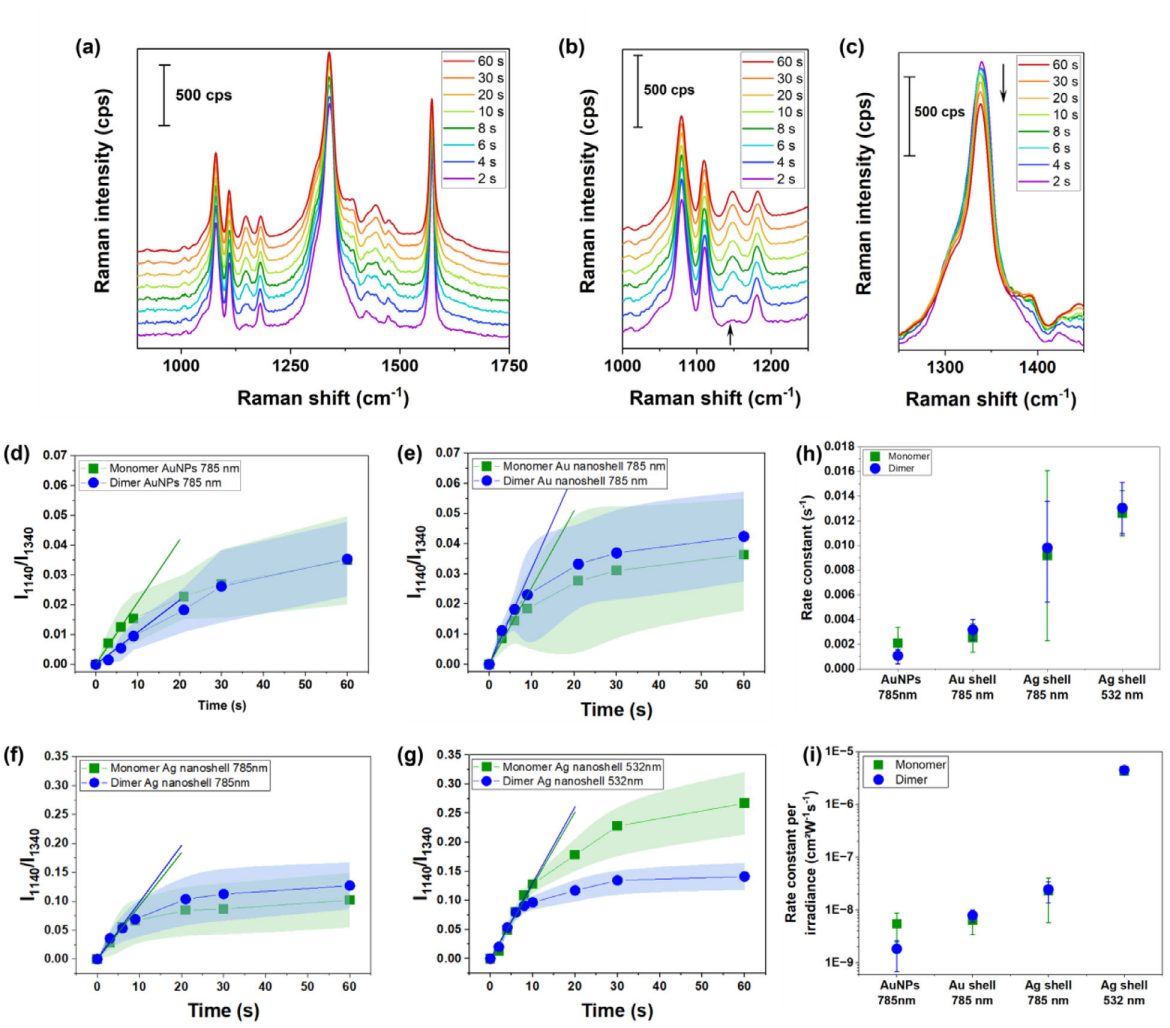
Plasmon‐induced p‐NTP conversion followed by Raman spectroscopy. (a) Typical Raman spectra showing the light‐induced conversion of p‐NTP after laser irradiation at 532 nm with a power of 0.1 mW for durations up to 60 s of laser exposure. The spectra were offset for clarity and obtained on the Ag nanoshell/SiNW dimer substrate. (b) Magnified offset spectra shown in (a) of the rising DMAB band at ca. 1140 cm^−1^. (c) Magnified spectra shown in (a) of the p‐NTP band at ca. 1340 cm^−1^ showing the consumption of the educt. (d–g) Time evolution of the ratio of the Raman band intensities at 1140 and 1340 cm^−1^ for each substrate investigated, as indicated in the Figure legends. Each point corresponds to the average intensity ratio obtained over 10 different substrate locations. The shaded colored regions show the standard deviation at each time point. Linear fits for determining the initial slope, and thus, reaction rate constants of the p‐NTP dimerization are given. (h) Reaction rate constants of the p‐NTP dimerization for each substrate. (i) Reaction rate constants normalized by laser irradiance of the p‐NTP dimerization for each substrate (logarithmic scale).

In a first set of experiments, the p‐NTP dimerization reaction was performed with a 785 nm laser, which provides higher SERS intensities on Au/SiNW arrays than shorter wavelengths, such as 532 nm, due to the larger E‐field enhancement generated at 785 nm (Figure 5). A relatively low laser power of 1 mW was used to acquire the Raman spectra of p‐NTP. In contrast, a much higher laser power of 30 mW was used to initiate the plasmon‐driven photodimerization of p‐NTP to form DMAB. This alternating sequence of Raman spectrum acquisition at 1 mW and catalytic reaction at 30 mW was repeated until a total irradiation duration of 60 s at 30 mW was reached.

In a second set of experiments, the influence of hot electron energy was investigated by comparing two different laser excitation wavelengths, i.e., 785 and 532 nm, on Ag nanoshell/SiNW arrays. Ag nanoshell/SiNW arrays were used for this experiment as these substrates still provide a significant near‐field enhancement at 532 nm, which enables us to directly probe the influence of excitation energy, and thus of hot electron energy. A minimum laser power of 0.1 mW was used in this case, since rapid dimerization of p‐NTP was observed at 532 nm. The underlying mechanisms responsible for this higher activity will be discussed later. DMAB formation and Raman spectra acquisition were conducted simultaneously at 532 nm to minimize measurement artifacts arising during Raman spectrum acquisition.

Figure 6a shows typical Raman spectra of these experiments, obtained on a Ag nanoshell/SiNW dimer array. The 1140 cm^−1^ band intensity assigned to DMAB increases with irradiation duration (Figure 6b), while the 1340 cm^−1^ band intensity of pNTP decreases (Figure 6c). The relatively low intensity of the DMAB band in comparison to the symmetric NO_2_ stretching of p‐NTP is consistent with previous observations and is attributed to the fact that not all available p‐NTP molecules are converted into DMAB, due to the non‐uniform spatial distribution of hotspots [9, 57]. Figure 6d–g compare the time evolution of I_1140_/I_1340_, which indicates the progress of the plasmon‐driven catalytic reaction, for all substrates. The reaction rate constant of the p‐NTP dimerization was estimated for each substrate (Figure 6h, see Section 4 and Equations S1–S7 for more details) and used to quantify the efficiency of the different substrates [7, 58]. The calculated reaction rate constants range from 0.00108 s^−1^ (AuNPs/SiNW dimer arrays) to 0.01305 s^−1^ (Ag nanoshell/SiNW dimer arrays at 532 nm), (see Figure 6h). These results broadly agree with literature values [23, 58, 59], although a direct comparison is not trivial because the rate constant depends on the excitation wavelength, the initial p‐NTP concentration, and irradiance at the substrate, which is directly influenced by experimental conditions such as laser power, objective numerical aperture, etc. Additionally, the method used to determine the rate constants is not always consistent across the literature [10, 23, 58, 59]. In this work, we systematically investigate a range of nanostructured substrates with distinct morphologies and compositions, all measured under identical experimental conditions. Despite the inherent challenges in accurately determining absolute rate constants, this approach enables a direct and robust comparison of photocatalytic performance across the different systems.

Interestingly, DMAB formation is observed on all nanostructured substrates, but is notably absent in the reference substrates, i.e., flat silicon coated with AuNPs or a Au‐sputtered film (Figure S4a,b). Additionally, Raman measurements of p‐NTP on bare SiNW monomer and dimer arrays did not yield any detectable Raman signal (Figure S4c). The negative reference measurements indicate that under our experimental conditions, AuNPs deposited on flat silicon cannot perform plasmon‐driven catalysis. However, when coupled with VA‐SiNW arrays, the AuNPs become photochemically active. The detection of the reaction product DMAB suggests that this activity results from interfacing the AuNPs with the VA‐SiNW arrays (Figure 5c,d), which significantly improves the generation of hot electrons within the AuNPs. The difference between flat substrates and SiNW arrays decorated with the same plasmonic elements demonstrates that VA‐SiNW arrays can accelerate plasmon‐driven reactions and are thus suitable substrates for plasmonic catalysis: When coated with small metal NPs, they provide robust E‐field‐enhancing capabilities.

We now take advantage of the systematic design of our hybrid platforms to compare the different samples and gain detailed insight into their plasmon‐enhanced catalytic behavior to elucidate structure–property relationships.

### 2.4 AuNPs vs. Au Nanoshells

Surprisingly, the AuNPs/SiNW substrates, which show the highest E‐field enhancement (see Figure 5), are slightly less efficient in their photocatalytic properties compared to the Au nanoshell/SiNW substrates (Figure 6d,e,h,i). This suggests that while the maximum E‐field determines the measured SERS intensity, it does not directly correlate with the efficiency of a substrate in plasmon‐driven catalysis [57]. We rationalize this discrepancy with geometrical considerations. The hotspots dominating the large SERS signal on the AuNPs/SiNW substrates are located at the AuNP/Si interface (Figure 5f,g). Due to the small radius of curvature of the AuNPs, these hotspots are small and highly confined between the two surfaces. This creates significant steric hindrance that limits the probability for two adjacent p‐NTP molecules to be appropriately bound to the Au surface with their nitro groups close to each other, which is the necessary requirement for the photodimerization reaction to form DMAB. Additionally, the reduced space available in the confined hot spot region might prevent the formation of the larger DMAB molecule. Similar observations have also been attributed by other groups to the limited geometrical accessibility of specific plasmonic hotspots [9, 57, 60]. This suggests a potential limitation of such metal NP/VA‐SiNW substrates for plasmon‐driven catalysis. In contrast, using Au and Ag nanoshells that conformally coat the entire VA‐SiNW produces less efficient local hotspots, but opens more space for the molecules to adsorb and react. Therefore, our results suggest that geometric effects dominate the overall reactivity.

### 2.5 Monomer vs. Dimer Arrays

Regardless of the geometry of metal nanostructures, the metal/VA‐SiNW dimer arrays, where pairs of individual nanowires are in close proximity, show no substantial improvement over the substrates with VA‐SiNWs with uniform spacing in either conversion efficiency or reaction rate (Figure 6d–i). This behavior contrasts with the SERS results, which for the SiNW dimer arrays consistently exhibited higher Raman signals (see Figure 4). This discrepancy further supports the hypothesis that the maximum local E‐field enhancement is not the main determining factor in plasmon‐driven catalysis. Our simulations show a significantly higher E‐field enhancement in the gap region of the SiNW dimers (Figure 5d,i,k,m) compared to the isolated SiNW monomers (Figure 5c,h,j,l). However, outside the gap region, the SiNW dimers exhibit slightly weaker field enhancement compared to the isolated SiNW monomers. Additionally, the large E‐field enhancement provided by the dimer arrays can only be reached when the light is polarized along the longitudinal axis of the dimer structure, which maximizes near‐field coupling in the gap region [61]. Finally, unlike the E^4^ dependency of SERS [62], hot electron generation scales as E^2^ [20], which makes it less sensitive to the nature of electromagnetic hotspots. Taken together, our experimental and simulation results suggest that, although the arrays of individual VA‐SiNWs do not enhance the E‐field as efficiently as the SiNW dimer arrays (visible in the SERS intensities, Figure 4), they provide a more uniform enhancement, which leads to comparable plasmon‐driven catalytic reaction rates.

### 2.6 Au Nanoshells vs. Ag Nanoshells

A comparison of the reaction rates between the Au nanoshell and Ag nanoshell/SiNW substrates clearly shows that the Ag nanoshell/SiNW arrays exhibit higher efficiency than the Au nanoshell/SiNW arrays (compare Figure 6e,f). We attribute this increased performance to the superior catalytic activity of Ag for p‐NTP photoreduction, consistent with previous reports showing higher efficiency of AgNPs relative to AuNPs [23]. The higher activity of Ag compared to Au originates from its more pronounced plasmon resonance and its higher Fermi level energy, which produces hot electrons with higher energies that can be injected more efficiently into the LUMO of p‐NTP and thus reduce it into DMAB [62]. Additionally, the greater tendency of silver to adsorb surface oxygen has been cited as a reason for its higher activity. Indeed, DFT calculations show that the dissociation of oxygen from the p‐NTP molecule, a necessary step in the photoreduction of p‐NTP, is energetically more favorable on silver than on gold, which explains the higher photoactivity of silver to form DMAB [22, 63]. The higher activity of Ag nanostructures is not only reflected in faster reaction rates, but is also observed in the final conversion after 60 s illumination, where Ag nanostructures reach higher conversions compared to Au nanostructures (compare I_1140_/I_1340_ in Figure 6d–i after 60 s and Figure S5). Notably, the reaction rate at our Ag nanoshell/SiNW substrates is approximately one order of magnitude larger than what has been measured on arrays of AgNPs separated with a 10 nm gap, i.e., ca. 0.001 s^−1^ at 785 nm (roughly extrapolated value to match our experimental conditions) [59], which further demonstrates the beneficial effect of using a supporting VA‐SiNW scaffold.

### 2.7 785 nm vs. 532 nm Laser Excitation

Finally, we investigated the influence of the excitation wavelength using the Ag nanoshell/SiNW arrays, which are photoactive at both 532 and 785 nm. A higher reaction rate was measured under excitation at 532 nm, although the incident laser power used was ca. 300 times lower than with a laser excitation wavelength of 785 nm. When normalized per incident irradiance, the reaction rate measured at 532 nm thus becomes remarkably large, with a two to four orders of magnitude increase compared to the other Au nanoshell/SiNW and Ag nanoshell/SiNW substrates at 785 nm laser excitation (Figure 6d–i). Additionally, the SERS intensity, which provides an experimentally averaged measure of the E‐field enhancement, is similar on these samples at both wavelengths (Figure 4b). Our results unambiguously demonstrate a higher plasmonic activity toward the reduction of p‐NTP on nanostructured Ag compared to nanostructured Au, and on the Ag surfaces, a much faster reaction at 532 nm laser excitation than at 785 nm laser excitation. This suggests that at 785 nm laser excitation, most hot electrons produced on an Ag surface lack the energy needed to trigger p‐NTP reduction, whereas at 532 nm laser excitation, a significant fraction exceeds this threshold energy. An energetic comparison can rationalize this hypothesis. The energy difference between the Ag Fermi level and the p‐NTP LUMO is between ca. 2 and 2.5 eV [64, 65, 66], which can be provided by electron excitation with a 532 nm laser (which corresponds to 2.33 eV), but not with a 785 nm excitation (which corresponds to 1.58 eV). At 532 nm, the Ag nanoshell/SiNW arrays show a higher conversion in comparison to 785 nm laser excitation, especially when normalized by the irradiance (see Figure 6d–i; Figure S5), which aligns with the higher reaction rates observed at this wavelength. Surprisingly, Ag nanoshell/SiNW monomer arrays show a larger overall conversion rate after 60 s than the Ag nanoshell/SiNW dimer array at 532 nm (compare I_1140_/I_1340_ in Figure 6g after 60 s and Figure S5), although both substrates provide similarly large reaction rate constants. Our FDTD simulations show that the integrated and normalized E^2^ enhancement on the entire metal surface is higher on the Ag nanoshell/SiNW monomer arrays compared to the dimer arrays: Although the hotspots produced by the Ag nanoshell/SiNW dimer arrays provide a locally higher absolute E‐field strength enhancement, the more regular E‐field strength enhancement generated by the Ag nanoshell/SiNW monomer arrays leads to the conversion of a higher number of p‐NTP molecules. Thus, we tentatively attribute the higher conversion measured on the monomer arrays to their more uniform density of moderate hotspots, which, at 532 nm, still provide sufficient hot electrons to form DMAB.

### 2.8 Reusability of Metal/SiNW Photocatalysts

The reusability of our metal/SiNW photocatalysts (AuNP coatings, Au nanoshell and Ag nanoshell) was investigated by performing 5 successive analyte deposition/photocatalytic reduction/O_2_ plasma cleaning cycles. Both AuNPs/SiNW and Au nanoshell/SiNW substrates could be successfully used for at least 5 cycles (Figure 7a). Interestingly, the efficiency of the Au nanoshell/SiNW sample significantly improved after each cycle: After the fifth cycle, the rate constantly increased by a factor of ca. 9. SEM analysis reveals that the initially smooth Au shell restructures and develops a higher roughness with some open pores (Figure 7b,c), expected to increase hot spot density, while geometrically favoring the dimerization reaction. This contrasts with the AuNPs/SiNW samples, which showed very stable photoactivity with a constant reaction rate during the 5 photocatalytic cycles.

**FIGURE 7:**
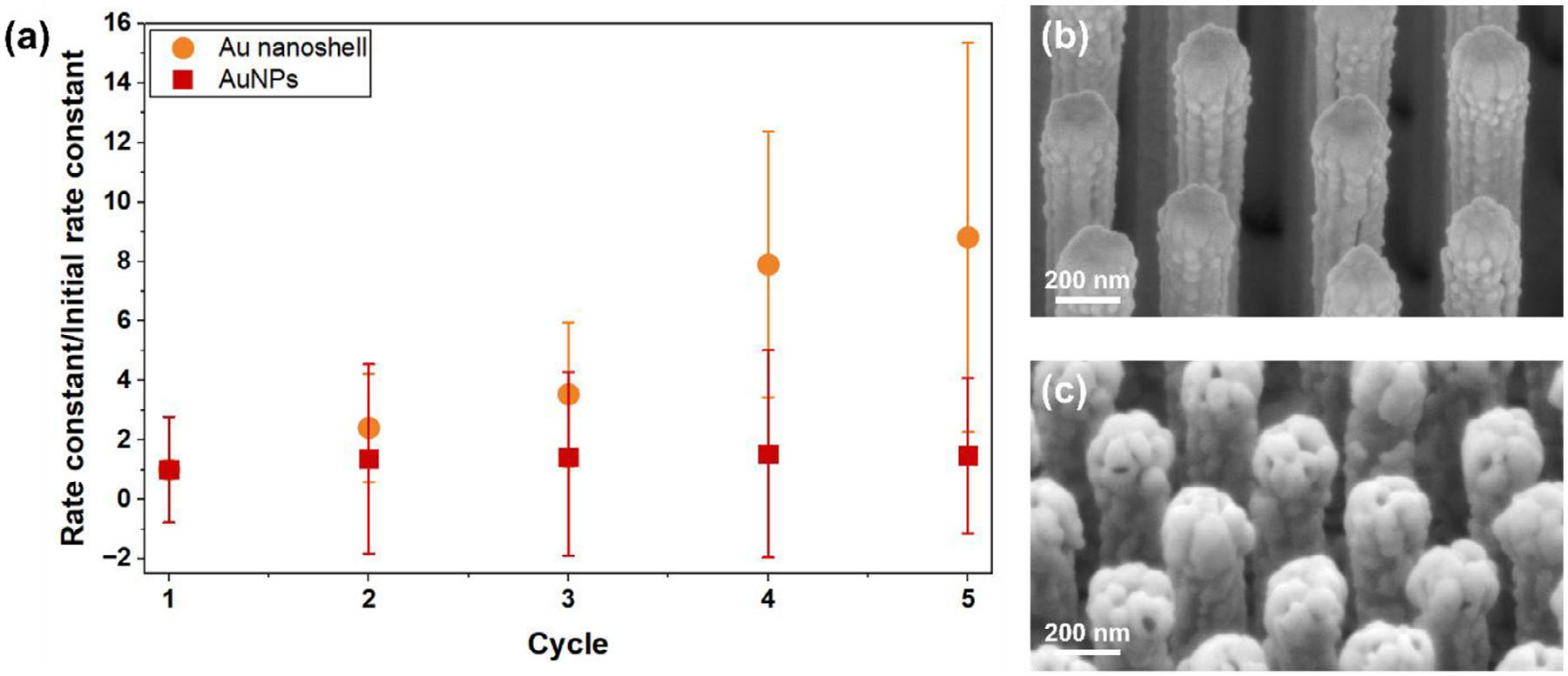
Reusability of metal/SiNW photocatalysts for p‐NTP photoreduction. (a) Ratio k_x_/k_1_ where k_x_ represents the rate constant after x photocatalytic p‐NTP reduction/cleaning cycles on Au nanoshell/SiNW monomer substrates (orange circles) and AuNPs/SiNW monomer substrates (red rectangles). (b, c) Tilted SEM images of the pristine Au nanoshell/SiNWs (b) and after five photocatalytic/cleaning cycles (c).

The Ag nanoshell/SiNW sample, on the other hand, did not withstand even weaker O_2_ plasma cleaning. After one cycle, the SERS response could not be re‐established, showing the difficulty in reusing such Ag‐based photocatalytic substrates.

Overall, our results demonstrate the following: (i) the AuNP coating provides the most stable substrates, (ii) the possibility to significantly increase the photoactivity of the Au nanoshell/SiNWs via successive O_2_ plasma treatment, and (iii) Ag nanoshell/SiNWs cannot be reused.

### 2.9 ROS‐Assisted Decarboxylation of 4‐Mercaptobenzoic Acid

To demonstrate the broader catalytic relevance of our hybrid substrates, the plasmon‐driven decarboxylation of 4‐mercaptobenzoic acid (4‐MBA) was performed on Ag nanoshell/SiNW arrays. At 532 nm, the hot electrons generated by Ag nanoparticles can reduce adsorbed molecular oxygen to generate superoxide radicals (i.e., O_2_ + e^−^ → O_2_˙^−^) [13]. Such ROSs have been shown to enable the oxidative decarboxylation of 4‐MBA into thiophenol [13]. After 2 min of 532 nm irradiation (10 mW power), a new Raman band emerges at ca. 999 cm^−1^, which corresponds to the in‐plane benzene ring bending δ_CCC_ of thiophenol (Figure S6) [13, 67]. 4‐MBA decarboxylation was not investigated on Au/SiNW substrates since it can only proceed effectively at 633 nm on Au surfaces, which is not available on our confocal Raman system [13]. Overall, the demonstrated ability of our metal/SiNW substrates to perform both reduction and ROS‐mediated oxidation reactions under light irradiation highlights their potential as multifunctional photocatalysts.

## 3 Conclusion

In this work, we investigate VA‐SiNW monomer and dimer arrays coated with AuNPs, Au nanoshells, and Ag nanoshells for SERS and hot electron‐induced reduction of p‐NTP. No p‐NTP conversion was observed on metal/flat silicon substrates, while all metal/VA‐SiNW arrays were able to produce DMAB, highlighting the E‐field‐enhancing abilities of VA‐SiNWs for plasmon‐driven catalysis. Previous studies on plasmon‐driven catalysis have shown that the dielectric environment of the metallic nanoparticle plays a decisive role in tuning local field enhancement and reaction rates, where high–refractive index substrates (e.g., TiO_2_, Cu_2_O, or Si_3_N_4_) [68, 69, 70] have been employed to improve light‐matter coupling. These demonstrations, however, have relied either on disordered dispersed heterostructures [68, 70] or planar substrates with limited surface area that restricts the available catalytic interface [69]. In contrast, our VA‐SiNW arrays provide not only a consistent E‐field enhancement for efficient near‐field coupling (see Figure 5), but also a large surface area scaffold that maximizes the density of accessible catalyst surface. As such, our substrates enable simultaneous enhancement of the local E‐field and increased catalytic site availability, distinguishing our platform from existing hybrid metal/high refractive index heterostructures for plasmon‐driven catalysis (Figures 5 and 6) [71, 72]. Additionally, VA‐SiNW arrays are also a suitable nanoscale template for the synthesis of metal nanoshell arrays that can generate surface E‐field enhancements that are appropriate for plasmonic catalysis (Figures 5 and 6).

Surprisingly, the substrates providing the largest SERS signal, i.e., the AuNPs/SiNW substrates, did not provide the fastest reaction rates, which we attribute to steric hindrance in the hot spot region. Our systematic study therefore suggests that geometric considerations are an important factor for plasmon‐driven photocatalytic conversions to synthesize large molecules. Additionally, Ag/SiNW arrays demonstrated significantly higher photoactivity at 532 nm excitation, which highlights the importance of selecting not only the appropriate photocatalyst, but also the proper photon energy.

Additionally, the ability of Ag nanoshell/SiNW substrates to drive ROS‐mediated oxidation under light irradiation highlights the broader potential of metal/SiNW hybrids for photocatalytic applications (Figure S6). Furthermore, the substantial enhancement in the photocatalytic activity of Au nanoshell/SiNW arrays, by up to a factor of nine after five photocatalytic/cleaning cycles (Figure 7a), demonstrates both the possibility to effectively reuse these substrates and the opportunity to further optimize their photocatalytic performance.

To the best of our knowledge, these research results represent the first demonstration that a high‐surface‐area nanostructured dielectric array, namely VA‐SiNW arrays, can enhance light–matter interactions to enable plasmon‐driven catalysis. Easy‐to‐prepare VA‐SiNWs can also be readily combined with a wide range of metal catalyst nanoparticles, provided these can be effectively immobilized on the nanowire surface. Although we have investigated comparably short VA‐SiNWs in this work, we expect our approach to be fully compatible with much longer VA‐SiNWs, which can be synthesized easily via MACE [73]. In addition, bulk silicon absorbs nearly 70% of incident solar energy, which optimizes photothermal effects under solar irradiation [74, 75]. Taken together, these attributes establish VA‐SiNWs as scalable, high‐surface‐area platforms with strong potential for plasmon‐driven catalysis in industrially relevant reactions, a direction we are currently investigating.

## 4 Experimental Section/Methods

### 4.1 Materials

All the chemicals mentioned here were used without further processing. APTES, HAuCl_4_·3H_2_O, iodine, potassium iodide, and anhydrous toluene (99.8%) were purchased from Sigma–Aldrich. Acetone (99%), calcium chloride, hydrofluoric acid (40%), isopropyl alcohol (≥ 98%), absolute ethanol (99.96%) and ethanol (96%) were ordered from VWR. Hydrogen peroxide (30%) and trisodium citrate dihydrate were acquired from Merck. Sulfuric acid (95%–97%) was supplied by Supelco. p‐NTP was purchased from Thermo Fisher Scientific. Double deionized Milli‐Q water with a resistivity of 18 MΩ from a Milli‐Q system was used. N‐doped Si wafers (<100>, resistivity 1–30 Ω cm) were purchased from Si Materials, Germany. 4‐MBA was purchased from Sigma–Aldrich, and isopropanol (≥98%) was purchased from VWR. SiO_2_@PDMAEMA core–shell particles used as a colloidal template were synthesized using materials as previously described [76].

### 4.2 Preparation of VA‐SiNW Monomer and Dimer Arrays

SiO_2_@PDMAEMA core–shell particles were synthesized as described elsewhere [37, 49, 76, 77, 78]. The utilized Si substrates were rinsed with ethanol and cleaned in an oxygen plasma for 5 min at 100 W (4 sccm, Femto SLS, Diener, Germany) in order to induce a hydrophilic surface. SiO_2_@PDMAEMA particles were mixed with absolute ethanol in a 1:1 ratio and assembled at the liquid‐water interface in a Langmuir‐Blodgett trough. The Si substrates were removed from the trough at a 90° angle. Particle dimers were achieved by compressing the core–shell particle layer by adjusting the surface pressure to a value of ca. 24 mN/m [49, 76, 79]. Subsequently, the polymer shell of the core–shell particles was entirely removed in an oxygen plasma treatment (50 W, 12 min, Quorum Emitech K1050X with an RF of 13.56 MHz, 20 mL/min). An adhesion layer of aluminum‐doped zinc oxide (AZO) was sputtered (75 W, 1 s, Clustex 100 M sputtering system by Leybold Optics) before a gold film was deposited (40 mA, 200 s, Cressington sputter coater 108 auto). The colloidal mask made up of the SiO_2_ spheres was lifted off using adhesive tape, leaving behind a gold nanohole film. This Au nanohole film was used as a catalytic etching mask for the subsequent MACE procedure. Before, the substrate was cleaned again in oxygen plasma for 5 min at 50 W. The samples were etched in the MACE solution (10 mL HF, 10 mL H_2_O, 750 µL H_2_O_2_) for the desired times (2, 4, or 6 min) and rinsed three times in deionized water. Finally, the samples were incubated for 5 min in an aqueous HF solution (4 mL HF, 20 mL H_2_O) to remove any porous SiO_2_ that formed on the surface of the SiNWs due to diffusion of holes. After washing the samples three times in deionized water and once in ethanol, they were dried in air. The catalytic Au film at the bottom of the SiNW array was removed via etching in an aqueous KI/I_2_ solution (85 g H_2_O, 10 g KI, 5 g I_2_) for at least 80 min, followed by two washing steps in deionized water and one washing step in ethanol [31, 37, 43, 48].

### 4.3 Synthesis of Au‐ and Ag‐SiNW Hybrid Structures

Au and Ag nanostructures were either produced via sputtering or via self‐assembly of AuNPs synthesized in solution. Sputtering was performed on the cleaned (OPE for 10 min at 50 W) SiNW substrates in the Cressington sputter coater 108 auto at 40 mA for 1000 s (producing a continuous Au nanoshell around the VA‐SiNWs with an average thickness of (17 ± 2) nm) or 500 s (producing a continuous Ag nanoshell around the VA‐SiNWs with an average thickness of (24 ± 2) nm comparable to the Au nanoshell). On top of the SiNWs, a Au or Ag cap, respectively, with a thickness of ca. 150 nm was formed. AuNPs that were used for the self‐assembly were synthesized following the well‐established citrate route [80, 81]. The surface of the VA‐SiNWs and also flat Si was functionalized with (3‐aminopropyl)triethoxysilane (APTES), as previously described and established in our group. The functionalized Si substrates were incubated for three days in a 1:1 mixture of AuNP solution and absolute ethanol and subsequently washed twice with deionized water [31, 37]. Scanning electron microscopy was used to identify regions with vertically aligned SiNW monomers and dimers on the sample surface. After this, samples were cut accordingly.

### 4.4 Raman Measurements

The employed Raman spectrometer was a dispersive Thermo DXR2 Raman microscope (Thermo, USA) combined with the confocal microscope BX41 by Olympus Corp. (Japan). For performing the in situ Raman experiments, a 1 mM solution of p‐NTP in absolute ethanol was prepared. Samples were first cleaned with a short oxygen plasma treatment (1 min at 20 W) and then incubated in 2 mL of the 1 mM p‐NTP ethanolic solution to form a self‐assembled monolayer of the p‐NTP on the Au/SiNW substrates. After 2 h of incubation, the samples were rinsed with ethanol to remove any unbound molecules. For the Ag nanoshells, another method of analyte deposition was used, since incubation in ethanolic p‐NTP solution changed the morphology of the deposited Ag nanoshells. Here, a 2 µL droplet of a 1 mM p‐NTP solution was applied to the substrates and measured after being dried in air.

The experiments with 785 nm laser excitation were conducted at two different laser powers. 1 mW was used to record the spectra, and the reaction of p‐NTP to DMAB was induced under 30 mW illumination for defined time intervals. Spectra were recorded with an exposure time of 2 s, 3 acquisitions, a 10× magnification objective with a numerical aperture of 0.25, a 50 µm slit aperture, and a full range grating. The spot diameters under the experimental conditions were given by the Raman instrument and turned out to be 3.1 µm (785 nm laser) and 2.1 µm (532 nm laser). Since the photodimerization of p‐NTP at 532 nm laser excitation on Ag surfaces was already induced at the lowest possible laser power of 0.1 mW, the experimental procedure was slightly varied, and exposure and measurement were combined. Spectra were recorded (and concomitantly the photoreaction was induced) at 0.1 mW for 1 s, 2 acquisitions, a 10× magnification objective with a numerical aperture of 0.25, a 25 µm slit aperture, and a full range grating. For all wavelengths, the reaction was induced, and spectra were recorded at 10 different locations of the samples.

The obtained spectra were averaged over the 10 different positions for each point in time and afterward underwent a peak deconvolution in the Raman shift range between 900 and 1600 cm^−1^, where the peak area of the Raman bands at ca. 1140 and ca. 1340 cm^−1^ were extracted and used for further evaluation. For this, the software OMNIC for dispersive Raman was used. The standard deviation of the band area was extracted by the OMNIC software after peak deconvolution of the standard deviation spectrum, calculated by the software from the 10 spectra measured at 10 different positions. According to the literature, and as described in detail in the Supporting Information, the reaction rate or rate constant of the p‐NTP photodimerization is proportional to the initial slope of the I_1140_/I_1340_ curves [23, 58, 59]. Those values were calculated by fitting a linear curve equation to the data points in the first 6 s of the experiment, i.e., the linear regime. Just for the AuNPs/SiNW dimer arrays, the range was increased to 9 s because of the low efficiency of this substrate, extending the linear regime. Error bars were obtained by applying the linear fit procedure to the minimum and maximum I_1140_/I_1340_ values, respectively. The as‐obtained reaction rate constants are shown in Figure 6 in this work, in order to compare the efficiency of monomer and dimer arrays with different metal nanostructures amongst each other [10, 58].

4‐MBA decarboxylation was performed as follows: a 2 µL droplet of a 1 mM 4‐MBA solution in isopropanol was drop cast on the Ag nanoshell/SiNW monomer array. Raman spectra were recorded at 532 nm excitation wavelength at 0.5 mW using a 50 µm slit aperture for an exposure time of 2 s and 3 acquisitions. A full‐range grating and a 10× magnification objective with a numerical aperture of 0.25 were used. Decarboxylation was carried out at 10 mW during 2 min while all other settings were kept unchanged.

Reusability experiments were performed by adding a cleaning step after each photocatalytic step, done in a plasma oven (Quorum Emitech K1050X) at 50 W in an O_2_ atmosphere for 10 min in order to completely remove the surface‐bound p‐NTP molecules.

### 4.5 SEM Imaging

SEM images were recorded on a Zeiss Ultra Plus 55 equipped with a field emission gun and Gemini lenses. The InLens secondary electron (SE) detector and the angle‐selective back‐scattered electron (AsB) detectors were used to gather information on topographical and compositional contrast, respectively. The freely available software ImageJ was used to analyze the SEM images regarding the geometrical parameters of the Au/Ag‐SiNW hybrid structures that were used in the FDTD simulations [82].

### 4.6 STEM‐EDX

For preparing the VA‐SiNW samples for Scanning Transmission Electron Microscopy (STEM) investigations, some part of the substrates was scratched with a cleaned tweezer. The tip of the tweezer was carefully moved across the TEM grid (Quantifoil holey carbon film on Cu 400 mesh) to disperse the SiNWs on its surface. The as‐prepared grids were cleaned in He plasma with a Zepto Plasma Cleaner (Diener) for 15 s at 20 W. STEM‐EDX maps were recorded using a cold field emission gun JEOL F200 at 200 kV accelerating voltage. The probe diameter and probe current were 0.16 nm and 0.1 nA. The EDX maps were recorded on a large windowless Centurio EDX detector by JEOL (100 mm^2^, 0.97 sr, energy resolution < 133 eV).

### 4.7 Electromagnetic FDTD Simulations

The electromagnetic response of the samples was simulated with the software Lumerical by Ansys Inc. (Canonsburg, USA) [54, 83]. In order to model Si, SiO_2_, Au, and Ag, the dielectric functions of the respective materials were used as they are implemented in the software. Anti‐symmetric and symmetric boundary conditions were used in the *x* and *y* directions, and perfectly matched layers (PML) boundary conditions in the *z* direction. In the simulations of the AuNP/VA‐SiNW arrays, periodic boundary conditions were used in the *x* direction because, due to the addition of the AuNP, the symmetry of the array broke down in the *x* direction. All simulated structures were placed in vacuum (*n* = 1). The SiNWs were modelled with a 2 nm thin SiO_2_ shell on their surface in order to take into account the oxide layer that is naturally growing on top of Si surfaces in air. The SiNW height was set to 870 nm, and the SiNW diameter was 150 nm. The pitch was 550 nm for the SiNW monomer array, and 650 nm for the dimer arrays. The pitch corresponds to the nanowire center‐to‐center distance for the monomer array, and to the dimer center‐to‐dimer center distance for the dimer array. A plane wave source emitting linearly polarized light was used to illuminate the simulated structures with light between 780 and 790 nm or 527 and 537 nm, respectively. Frequency‐domain field monitors (DFT monitors) were used to investigate the E‐field distribution on the simulated structures. A mesh size of 1 nm (2D) was used around the AuNPs and a mesh size of 0.5 nm at the interface between AuNPs (2D) and SiNWs. AuNPs on flat Si were modelled with a 0.5 nm (2D) mesh, and the mesh size around the SiNWs and the metal nanoshells was 2 nm (2D). For integration of the E^2^ on the surface of the simulated nanostructures, a 2 nm shell with a refractive index of 1.00001 (similar to vacuum) was modelled around the nanostructures. The script contained in the Advanced Power Analysis group in the Lumerical software was adapted to obtain and integrate the (E‐field strength)^2^ components over the volume of the 2 nm shell [31, 37, 84].

## Author Contributions

**Theresa Bartschmid**: conceptualization, investigation, writing – original draft, methodology, validation, visualization, writing – review and editing, software, formal analysis, data curation. **Gilles Bourret**: conceptualization, investigation, funding acquisition, writing – original draft, methodology, validation, writing – review and editing, formal analysis, project administration, supervision, resources. **Furkan Atalay**: investigation. **Nicolas Vogel**: funding acquisition, writing – review and editing, validation, formal analysis. **Johannes Menath**: investigation. **Amin Farhadi**: investigation. **Maurizio Musso**: writing – review and editing, formal analysis. **Maret Ickler**: investigation.

## Funding

This research was funded in whole or in part by the Austrian Science Fund (FWF) DOI:10.55776/P33159. For open access purposes, the author has applied a CC BY public copyright license to any author accepted manuscript version arising from this submission. T.B. gratefully acknowledges the Austrian Academy of Sciences (ÖAW) for a full doctoral scholarship (DOC scheme), grant number 26939. N.V. acknowledges support from the German Science Foundation (DFG) under grant number VO1824/13‐1.

## Conflicts of Interest

The authors declare no conflicts of interest.

## Supporting information




**Supporting File**: smll74983‐sup‐0001‐SuppMat.pdf.

## Data Availability

The data that supports the findings of this study are available in the supplementary material of this article.
