# Supplementary material for: Metal/Si Nanowire Arrays for Hot Electron Catalysis
Source: Small. 2026 Aug 2;22(55):e74983. doi: 10.1002/smll.74983 (PMC13630402; doi:10.1002/smll.74983)
Supplement: Supplementary file 1 [file SMLL-22-e74983-s001.pdf]

SUPPORTING INFORMATION

Metal/Si Nanowire Arrays for Hot Electron Catalysis

*Theresa Bartschmid, Furkan Atalay, Maurizio Musso, Amin Farhadi, Johannes Menath, Maret Ickler, Nicolas Vogel, Gilles Bourret**

Theresa Bartschmid, Furkan Atalay, Maurizio Musso, Amin Farhadi, Gilles Bourret
Department of Chemistry and Physics of Materials, University of Salzburg, Salzburg, Austria
E-mail: gilles.bourret@plus.ac.at

Johannes Menath, Maret Ickler, Nicolas Vogel
Institute of Particle Technology, Friedrich-Alexander University Erlangen-Nürnberg, Erlangen, Germany

Funding: GB and TB acknowledge the Austrian Science Fund (FWF) for project P-33159. TB gratefully acknowledges the Austrian Academy of Sciences (ÖAW) for a full doctoral scholarship (DOC scheme), grant number 26939. NV acknowledges support from the German Science Foundation (DFG) under grant number VO1824/13-1.

Keywords: plasmon-driven catalysis, hot electron, SERS, silicon nanowires, Au nanoparticles, metal-assisted chemical etching

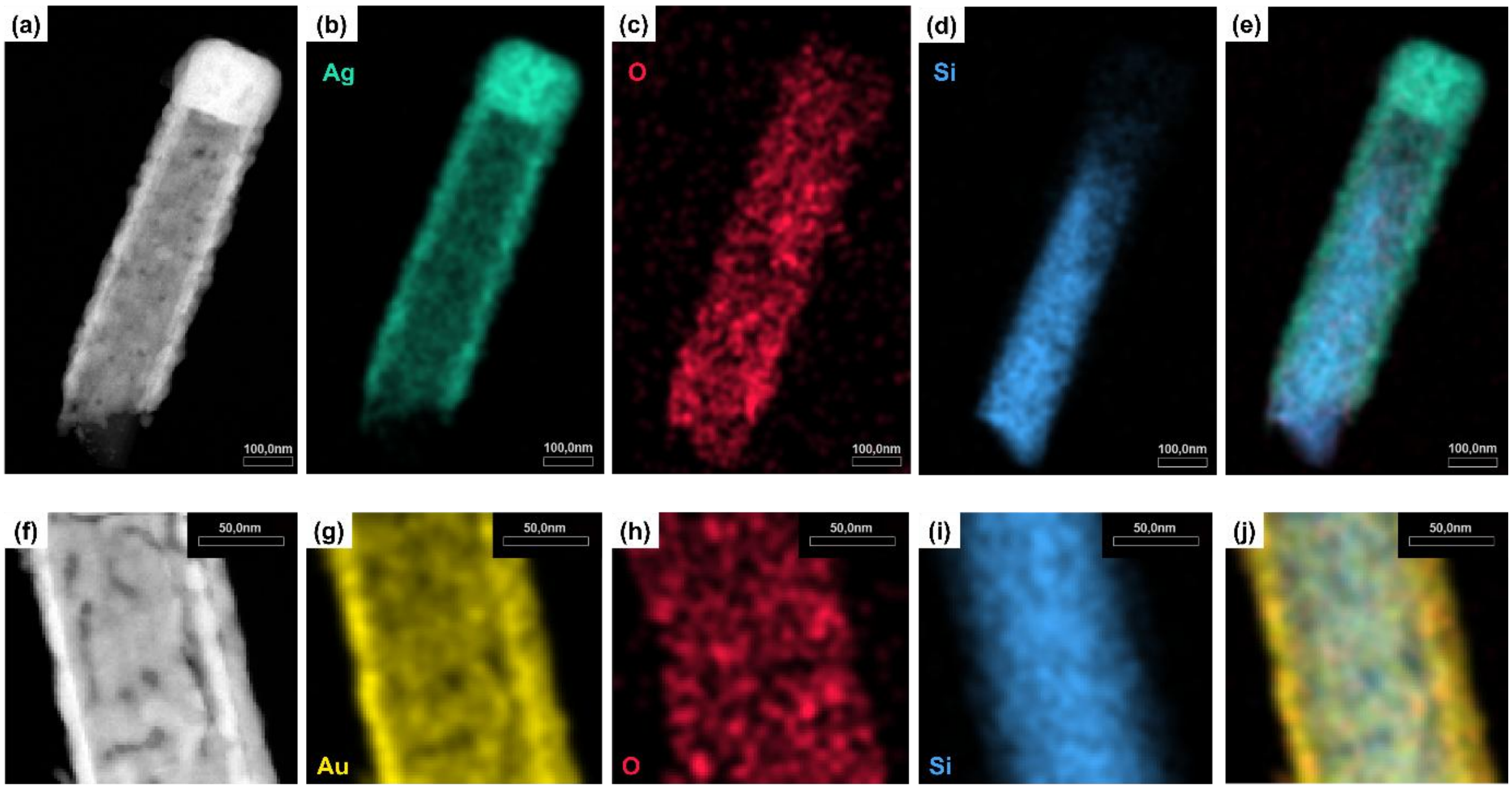

Figure S1. STEM-EDX maps of a SiNW coated with (a-e) a Ag shell (500 s sputtering) and (f-j) a Au shell (1000 s sputtering). (a, f) High-angle annular dark field images. (b, g) Silver and gold EDX maps. (c, h) Oxygen EDX maps. (d, i) Silicon EDX maps. (e, j) Overlay EDX maps.

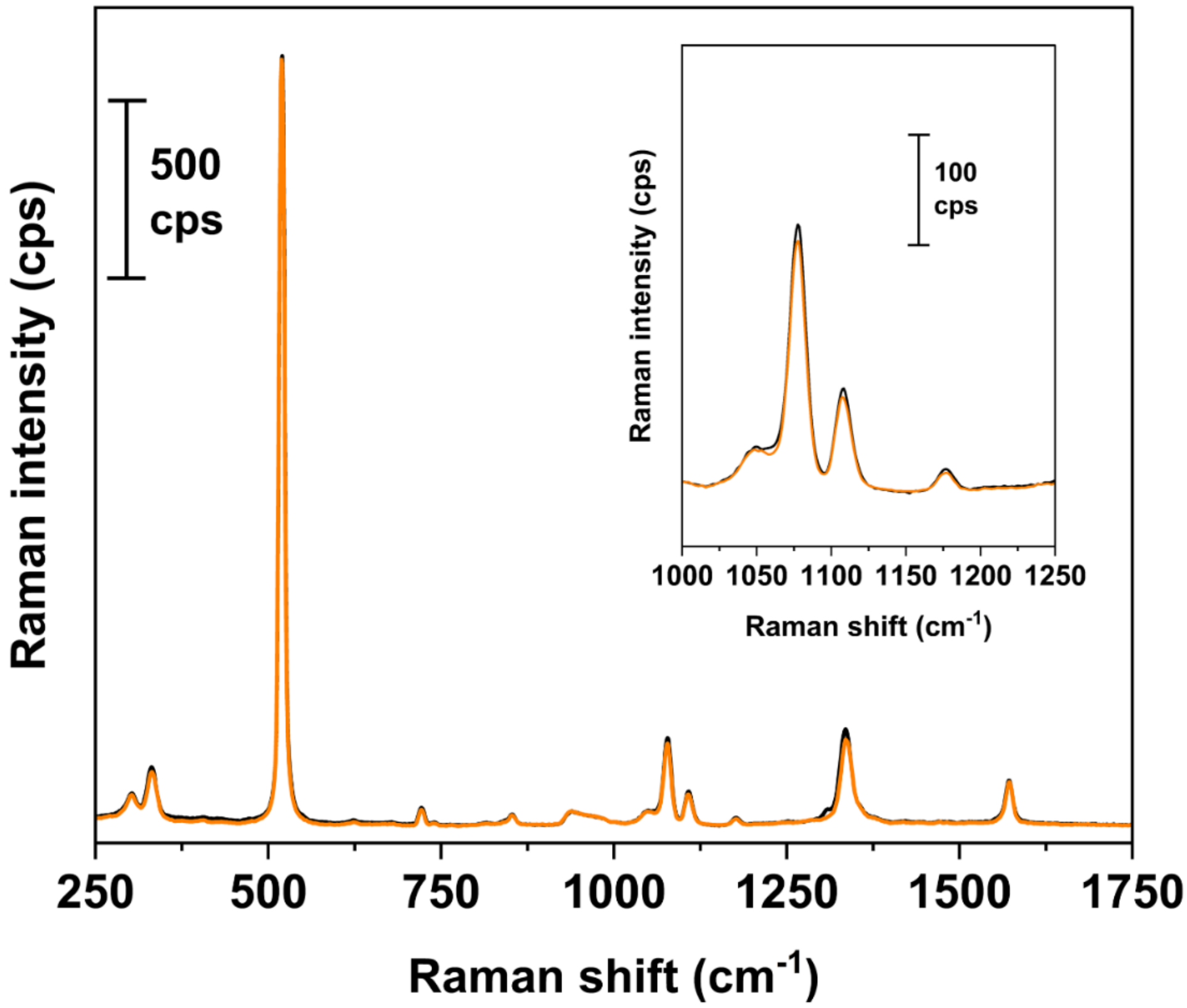

Figure S2. Raman spectra recorded after 0 s of illumination (black) and after 30 min of continuous laser illumination at 1 mW (orange) demonstrating that at this low laser power used for recording the Raman spectra shown in this work, no measurable DMAB formation is induced. This experiment was performed on SiNWs decorated with AuNPs.

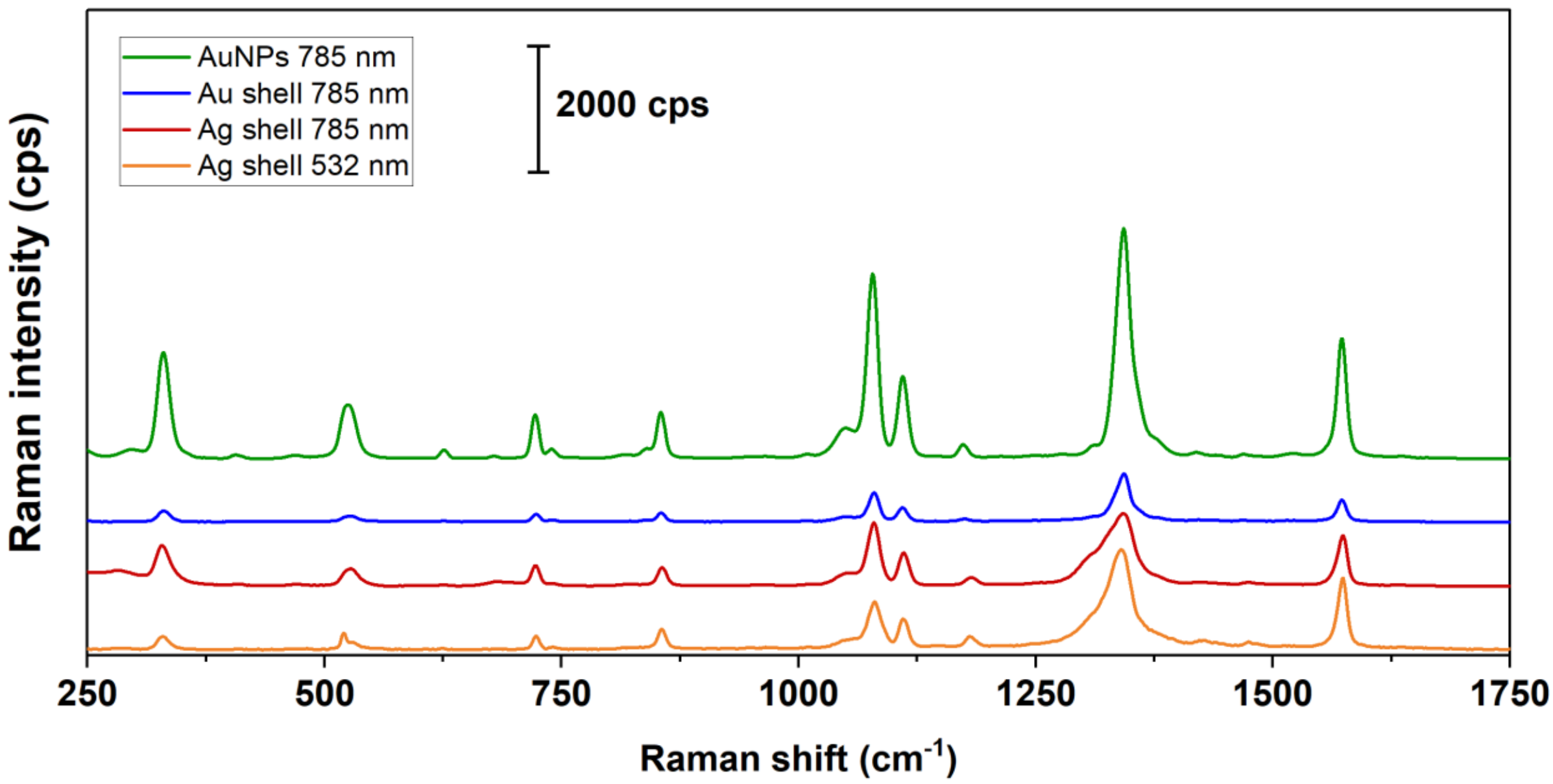

Figure S3. Offset SERS spectra recorded on SiNW monomer array substrates with different metal nanostructures as indicated in the Figure.

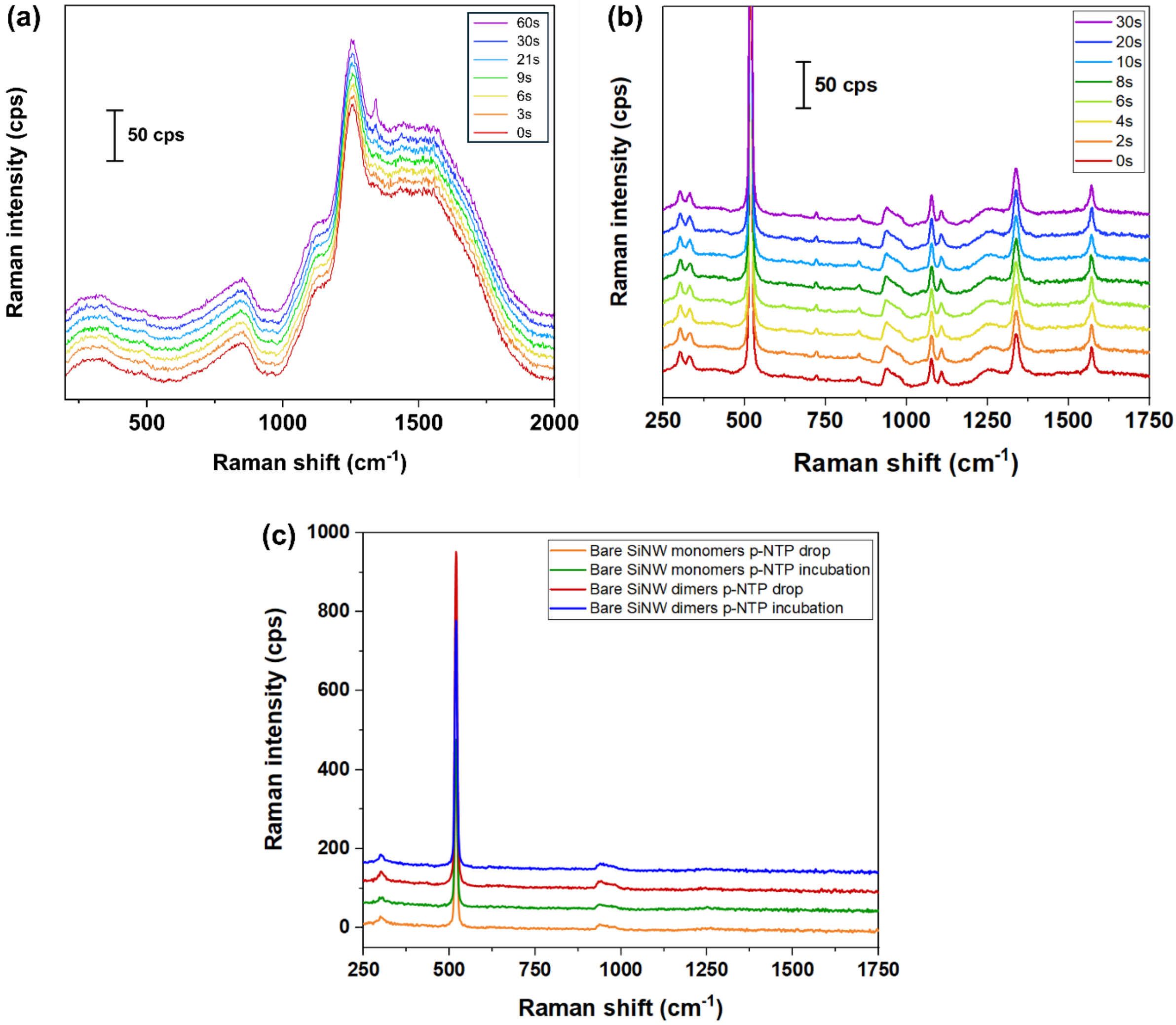

Figure S4. Raman spectra of flat Si decorated with (a) a Au layer obtained after 1000 s of sputtering and (b) self-assembled AuNPs showing no development of DMAB after 60 s or 30 s, respectively. (c) Raman spectra of bare SiNW monomer and dimer arrays without metallic nanostructure showing no detectable signal intensity of p-NTP, whether it was deposited *via* incubation or *via* drop-casting.

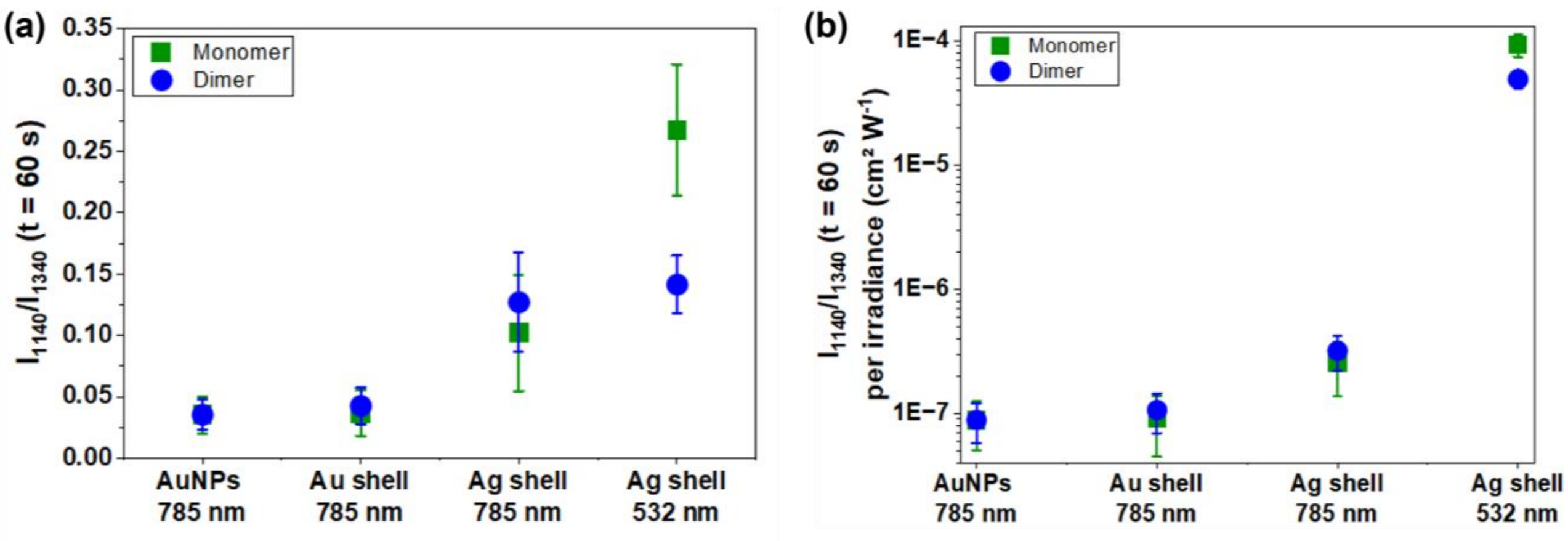

Figure S5. (a) Ratio of the Raman bands at 1140 cm^{-1} and 1340 cm^{-1} after 60 s laser exposure acting as a measure for the final conversion and (b) ratio of the Raman bands at 1140 cm^{-1} and 1340 cm^{-1} after 60 s laser exposure normalized per laser irradiance.

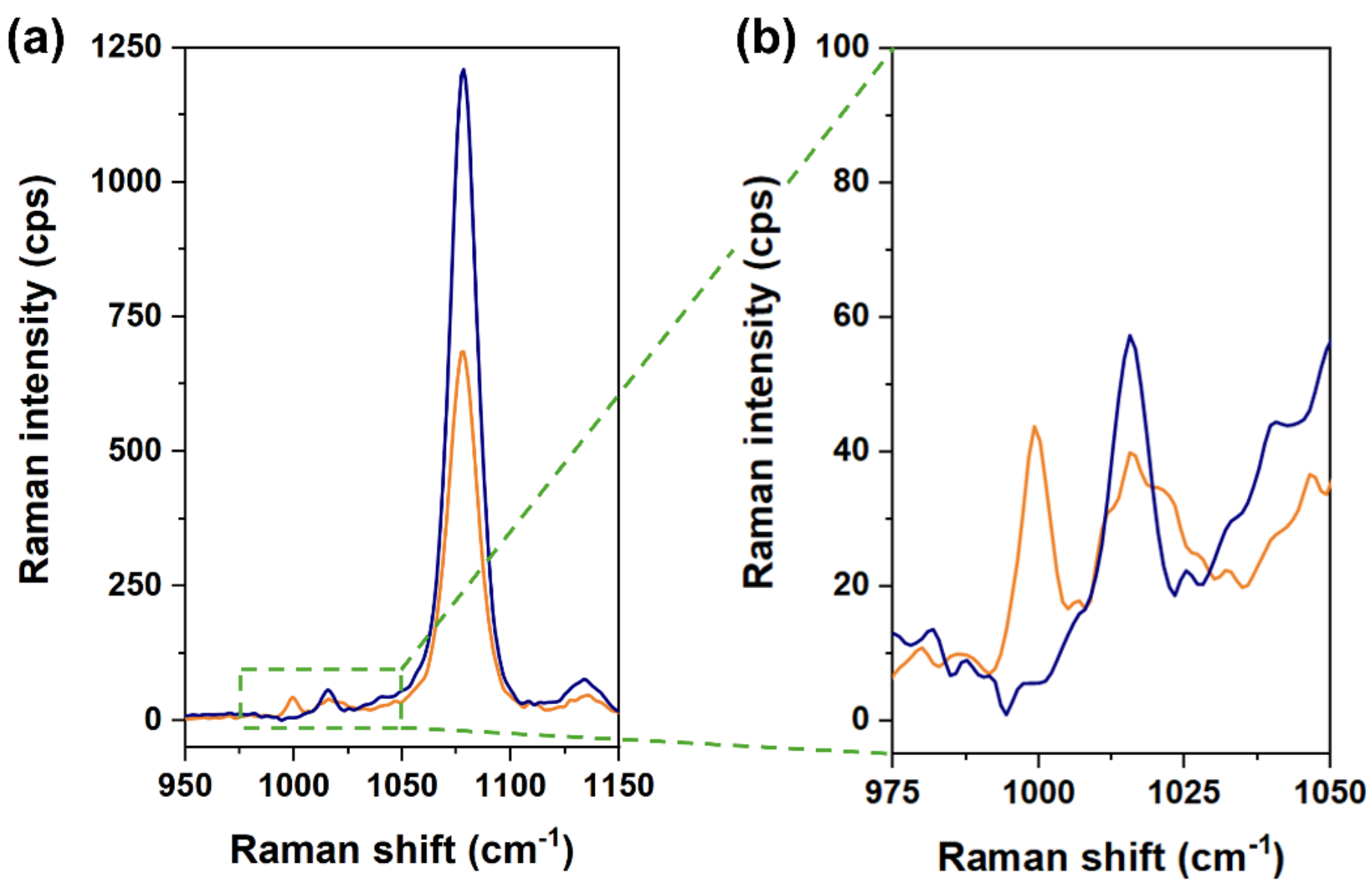

Figure S6. ROS-mediated 4-MBA decarboxylation. (a) Raman spectra of 4-MBA on a Ag nanoshell/SiNW monomer array before (blue) and after (orange) 532 nm exposure for 2 min at 10 mW, showing the emergence of a new band at ca. 999 cm^{-1}, characteristic of thiophenol formation due to the ROS-mediated decarboxylation of 4-MBA. (b) Magnified part of the spectrum showing the new band at ca. 999 cm^{-1}, characteristic of thiophenol formation.[1]

Calculation of reaction rate constants. For the calculation of reaction rate constants it was assumed that the p-NTP dimerization (2 p-NTP → DMAB) follows a second order reaction, as previously described in literature. The description here is mainly based on Koopmann et al. and Li et al.[2, 3]

Thus, the following differential equation is valid, where $[pNTP]$ and $[DMAB]$ are the concentrations of the reactant pNTP and the product DMAB, respectively, at a specific time t:

$$-\frac{d[pNTP]}{dt} = 2\frac{d[DMAB]}{dt} = k \cdot [pNTP]^2 \qquad \text{(S1)}$$

When integrated between t = 0 s and t and using $[pNTP]_{t=0s} = [pNTP]_0$, this differential equation can be solved to:

$$\frac{1}{[pNTP]_0} - \frac{1}{[pNTP]} = k \cdot t \qquad \text{(S2)}$$

$$\leftrightarrow \qquad [pNTP] = \frac{[pNTP]_0}{1+[pNTP]_0 \cdot k \cdot t} \qquad \text{(S3)}$$

Thus, the product Raman band intensity at a specific time t (I_{1140}), is related to the product concentration a specific time t ($[DMAB]$) and the Raman scattering cross section σ_{1140}:

$$I_{1140} = \sigma_{1140} \cdot [DMAB] = \frac{\sigma_{1140}}{2} \cdot ([pNTP]_0 - [pNTP]) = \frac{\sigma_{1140}}{2} \cdot \frac{([pNTP]_0^2 \cdot k \cdot t)}{1+[pNTP]_0 \cdot k \cdot t} \qquad \text{(S4)}$$

With this, the Raman intensity for the educt peak can similarly be described and we can then derive the time dependence of $\frac{I_{1140}}{I_{1340}}$:

$$\frac{I_{1140}}{I_{1340}} = \frac{1}{2} \cdot \frac{\sigma_{1140}}{\sigma_{1340}} \cdot [pNTP]_0 \cdot k \cdot t \qquad \text{(S5)}$$

which can be rewritten as:

$$\frac{I_{1140}}{I_{1340}} = k_2 \cdot t \qquad \text{(S6)}$$

with,

$$k_2 = \frac{1}{2} \cdot \frac{\sigma_{1140}}{\sigma_{1340}} \cdot [pNTP]_0 \cdot k \qquad \text{(S7)}$$

where k_2 can be considered as a rate constant and used to compare the dimerization kinetics on the different samples. Equations S5-S7 are only valid in the early stages of the reaction, where a linear regime can be clearly seen. Quickly, the reaction rate decreases as the dimerization reaches a plateau, which is due the occurrence of a dissociative side reaction of p-NTP, and the fact that a given fraction of p-NTP do not react.[4] There, the reaction rate k_2 was calculated using a linear fit for the first 6 seconds. For the AuNPs/SiNW dimer arrays, the first 9 seconds were used due to the low reaction rate for this substrate and thus, its longer linear regime.

References

1. Wei, Y.; Fan, X.; Chen, D.; Zhu, X.; Yao, L.; Zhao, X.; Tang, X.; Wang, J.; Zhang, Y.; Qiu, T.; Hao, Q., Probing Oxidation Mechanisms in Plasmonic Catalysis: Unraveling the Role of Reactive Oxygen Species. *Nano Lett.* **2024,** *24* (6), 2110-2117. DOI 10.1021/acs.nanolett.3c04979.

2. Koopman, W.; Titov, E.; Sarhan, R. M.; Gaebel, T.; Schürmann, R.; Mostafa, A.; Kogikoski Jr., S.; Milosavljević, A. R.; Stete, F.; Liebig, F.; Schmitt, C. N. Z.; Koetz, J.; Bald, I.; Saalfrank, P.; Bargheer, M., The Role of Structural Flexibility in Plasmon-Driven Coupling

Reactions: Kinetic Limitations in the Dimerization of Nitro-Benzenes. *Adv. Mater. Interfaces* **2021,** *8* (22), 2101344. DOI https://doi.org/10.1002/admi.202101344.
3. Li, P.; Ma, B.; Yang, L.; Liu, J., Hybrid single nanoreactor for in situ SERS monitoring of plasmon-driven and small Au nanoparticles catalyzed reactions. *Chem. Commun.* **2015,** *51* (57), 11394-11397. DOI 10.1039/C5CC03792A.
4. Koopman, W.; Sarhan, R. M.; Stete, F.; Schmitt, C. N. Z.; Bargheer, M., Decoding the kinetic limitations of plasmon catalysis: the case of 4-nitrothiophenol dimerization. *Nanoscale* **2020,** *12* (48), 24411-24418. DOI 10.1039/D0NR06039A.
